# Epithelial cell-derived micro RNA-146a generates interleukin-10-producing monocytes to inhibit nasal allergy

**DOI:** 10.1038/srep15937

**Published:** 2015-11-03

**Authors:** Xi Luo, Miaomiao Han, Jianqi Liu, Yu Wang, Xiangqian Luo, Jing Zheng, Shuai Wang, Zhigang Liu, Dabo Liu, Ping-Chang Yang, Huabin Li

**Affiliations:** 1 Department of Otolaryngology, Affiliated Guangzhou Women and Children’s Medical Center, Guangzhou Medical University, Guangzhou, China; 2 Department of Otolaryngology, Head and Neck Surgery, Xinhua Hospital, Shanghai Jiaotong University School of Medicine, Shanghai, China; 3 Allergy & Immunology Center, Shenzhen University School of Medicine, Shenzhen, China; 4 ENT Institute, Longgang Central Hospital, Shenzhen, China

## Abstract

The aberrant immunity plays an important role in the pathogenesis of allergic diseases. Micro RNAs (miR) are involved in regulating the immunity in the body. This study aims to test a hypothesis that miR-146a induces the expression of interleukin (IL)-10 in monocytes (Mos). In this study, the levels of miR-146a were determined by real time RT-PCR. The IL-10^+^ Mos were evaluated by flow cytometry. The miR-146a-laden exosomes were generated with RPMI2650 cells (an airway epithelial cell line). An allergic rhinitis mouse model was developed. The results showed that nasal epithelial cells expressed miR-146a, which was markedly lower in the nasal epithelial cells of patients with nasal allergy than that in healthy controls. Exposure to T helper (Th)2 cytokines suppressed the levels of miR-146a in the nasal epithelial cells. The nasal epithelial cell-derived miR-146a up regulated the expression of IL-10 in Mos. The inducible IL-10^+^ Mos showed an immune suppressor effect on the activities of CD4^+^ effector T cells and the Th2 polarization in a mouse model of allergic rhinitis. In summary, nasal epithelial cells express miR-146a, the latter is capable of inducing IL-10 expression in Mos, which suppress allergic reactions in the mouse nasal mucosa.

Allergic diseases affect most organs in the body. The aberrant T helper (Th)2 polarization plays an important role in the pathogenesis of allergic disorders. The Th2 responses include a higher frequency of Th2 cells and higher levels of Th2 cytokines, including interleukin (IL) -4, IL-5 and IL-13^1^, in the local tissue. Th2 cytokines up regulate the production of immunoglobulin (Ig) E; IgE sensitizes the allergic effector cells, including mast cells and eosinophils. Upon re-exposure to specific antigens, sensitized mast cells or/and eosinophils release allergic mediators to induce allergic inflammation^2^,^3^. To date, the therapeutic effect of allergic diseases is unsatisfactory currently^4^.

A fraction of cells has immune regulatory functions, which are designated immune regulatory cells, mainly including regulatory T cells (Treg)^5^ and regulatory B cells (Breg)^6^. The immune regulatory cells suppress the activities of other immune effector cells to prevent aberrant immune responses^5^,^6^. Apart from the natural Tregs from the thymus, Tregs can be induced in the peripheral system. A number of approaches have been reported in the generation of Tregs and Bregs, such as transforming growth factor (TGF)-β in the generation of Tregs^7^,^8^. The thrombospondin 1 is linked to the generation of Bregs^9^. In addition to Tregs and Bregs, other cell types are also involved in the immune regulation; such as alternatively activated macrophages have immune regulatory properties^10^. Macrophages are differentiated from monocytes (Mos). Myeloid-derived suppressor Mos are reported in the tumor tissue^2^. Factors inducing Mos to become regulatory cells are to be further investigated.

A micro RNA (miR) is a small non-coding RNA molecule (about 22 nucleotides). miRs function via base-pairing with complementary sequences within mRNA molecules^11^. They act by repressing target gene expression via targeting on the 3′-untranslated region (UTR) of mRNAs to induce either mRNA degradation or translation inhibition, or both^12^. miR-146a is one of the two members of the miR-146 miR family. miRs are involved in a number of cell activities; such as suppressing cancer growth^13^, inhibiting inflammation^14^, and suppressing allergic inflammation^15^. The increase in miR-146a in monocytes in response to microbial products, such as lipopolysaccharide (LPS) has been reported^16^. Based on the above information, we hypothesize that the miR-146a modulates naïve Mo properties to generate the regulatory Mos. To test the hypothesis, we carried out this study. The results showed that nasal epithelial cells produced miR-146a; the latter induced naïve Mos to produce interleukin (IL)-10. The IL-10-producing Mos were capable of suppressing effector T (Teff) cell activities and inhibiting nasal allergy in a mouse model.

## Results

### Nasal epithelial cells express miR-146a that is suppressed by allergic responses

**Figure 1:**
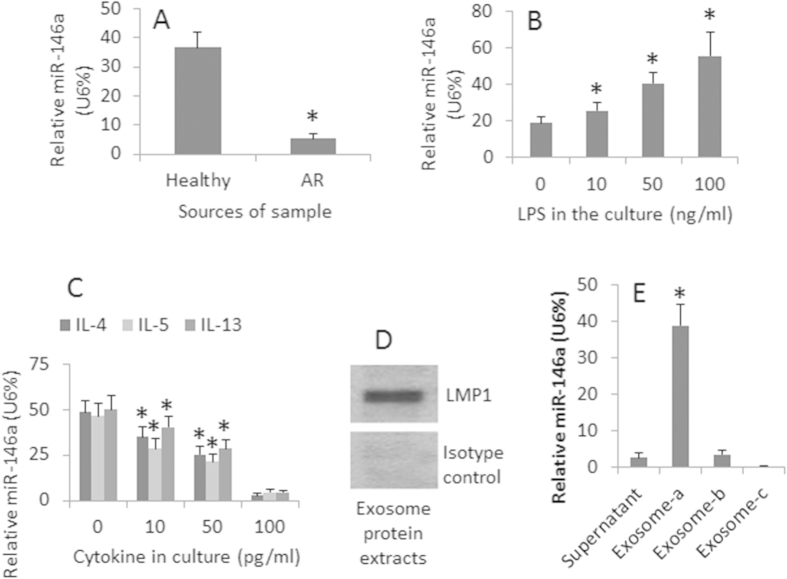
Assessment of miR-146a in the nasal epithelial cells. (**A**) human nasal epithelial specimens were scrapped from 10 healthy subjects and 10 patients with allergic rhinitis (AR). (**B**,**C**) RPMI2650 cells were cultured in the presence of LPS (B) or Th2 cytokines (**C** as denoted on the X axis) for 48 h. The total RNA was extracted from the RPMI2650 cells and analyzed by RT-qPCR. D-E, exosomes were purified from the RPMI2650 cell culture supernatant. Total proteins and RNA were extracted from the exosomes. (**D**) the Western blots indicate the LMP1 protein (a marker of exosomes). The gel below the Western blots is an isotype control. (**E**) the bars indicate the miR-146a levels in the supernatant, or exosomes (a: RPMI2650 cells were primed with LPS; b: naïve RPMI2650 cells; c: RPMI2650 cells were treated with the antisense of miR-146a). The bars indicate the levels of miR-146a (mean ± SD). *p < 0.01, compared with the healthy group (**A**), or dose “0” group (**B**,**C**), or supernatant (**E**). The data are a representative of 3 independent experiments.

In the first step, we assessed the levels of miR-146a in the human nasal epithelial cells. The results showed that miR-146a was detected in the nasal epithelial specimens obtained from healthy subjects, which was much lower in the patients with allergic rhinitis than that in the healthy subjects (Fig. 1A). We also detected the expression of miR-146a in RPMI2650 cells (an airway epithelial cell line). After exposure to LPS in the culture, the levels of miR-146a were markedly increased in a LPS dose-dependent manner (Fig. 1B). In separate experiments, RPMI2650 cells were exposed to Th2 cytokines (IL-4, IL-5 and IL-13) and LPS in the culture for 48 h, the expression of miR-146a was significantly suppressed in RPMI2650 cells (Fig. 1C). Based on published data that miRs can be enclosed in exosomes to be released out cells^17^, we purified exosomes from the culture supernatant of RPMI2650 cells. As shown by RT-qPCR data, after exposure to LPS, high levels of miR-146a were detected in the exosomes purified from the supernatant (Fig. 1D,E). The results suggest that human nasal epithelial cells produce miR-146a, which can be up regulated by LPS and suppressed by Th2 cytokines. The miR-146a can be released to the microenvironment carried by exosomes. We also assessed the levels of miR-155 and miR-125b in the exosomes. The results showed that the levels of miR-155 and miR-125b were below the detectable levels (Fig. S1 in the supplemental materials).

### Epithelial cell-derived miR-146a induces IL-10 expression in Mos

**Figure 2:**
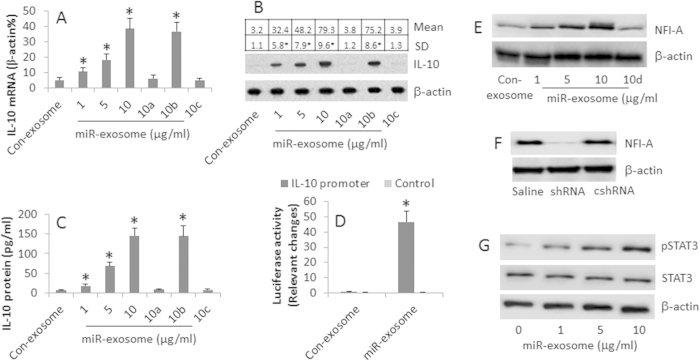
MiR-146a induces IL-10 expression in Mos. The CD14^+^ CD16^−^ Mos (Mo) were isolated from PBMC of healthy subjects and cultured for 6 days in the presence of miR-exosomes (miR-146a-laden exosomes) or con-exosomes (con-exosomes ; miR-146a-null) at doses as denoted on the X axis and IL-7 (10 ng/ml). (**A**) the bars indicate the mRNA levels of IL-10 in Mos (Assessed by RT-qPCR). (**B**) the Western blots indicate the protein levels of IL-10 in Mos. The table above the blots indicate the integrated density of the blots. (**C**) the bars indicate the protein levels of IL-10 in culture supernatant (Assessed by ELISA). (**D**) the bars indicate the IL-10 promoter activities assessed by luciferase reporter gene assay (IL-10 promoter: The cells were transduced with IL-10 promoter-luciferase gene constructs; control: The cells were transduced with control constructs). 10a: The “a” indicates that NFI-A gene was knocked down in the Mos. 10b: The “b” indicates that Mos were treated with control shRNA. 10c, the “c” indicates the presence of antisense of miR-146a in the culture (10 μg/ml). (**E**) the Western blots show the NFI-A levels in Mos after exposure to miR-146a-exosomes. 10d: The “d” indicates the presence of STAT3 inhibitor, Stattic (25 μM). (**F**) the Western blots show the NFI-A gene knockdown results. The Mos were treated with saline, or shRNA of NFI-A (shRNA), or control shRNA (cshRNA). (**G**) the Western blots show the levels of STAT3 and phosphorylated STAT3 (STAT3-p) in Mos after exposure to exosomes. The data of (**A**–**D**) are presented as mean ± SD. *p < 0.01, compared with the con-exosomes group. The data are a representative of 3 independent experiments.

Mos are one of the major components of leukocytes and distribute to all the parts of the body, including the subepithelial region of the mucosa. Published data indicate that miR-146a is involved in the suppression of inflammation^18^; Mos express lactoferrin receptor, a target of miR-146a^18^,^19^. We hypothesize that miR-146a regulates the properties of Mos, the latter further suppress immune inflammation. To test this, we isolated the CD14^+^ CD16^−^ Mos (the classic Mos) from PBMCs of healthy subjects. The cells were cultured in the presence of the miR-146a-laden exosomes (miR-exosomes) at gradient doses for 6 days. The results showed that exposure to miR-exosomes markedly increased the expression of IL-10 in the Mos at both mRNA and protein levels in a miR-exosomes dose-dependent manner (Fig. 2A,B). The Mo-derived IL-10 was also found in the culture supernatant (Fig. 2C). To strengthen the data, we transduced an IL-10 promoter-luciferase reporter gene to Mos; the Mos were exposed to miR-exosomes or con-exosomes in the culture. Exposure to miR-exosomes markedly increased the IL-10 promoter activity (Fig. 2D) as well as the IL-10 promoter methylation (Fig. S2). The results suggest that the airway epithelial cell-derived miR-146a induces naïve Mos to the IL-10-producing Mos. To enforce the results, in separate experiments, we added an antisense of miR-146a to the culture together with the same condition above. The results showed that the presence of the antisense of miR-146a abolished the miR-146a-induced IL-10 expression in Mos (Fig. 2A–D; Fig. S2). Since the type I regulatory T cells (Tr1 cells) also express IL-10, we wondered if the miR-exosomes could induce the IL-10-producing Tr1 cells. To this end, we cultured naive CD4^+^ T cells in the presence of the miR-exosomes. But no detectable CD4^+^ IL-10^+^ T cells were induced (Fig. S3).

Published data indicate that nuclear factor one A (NFI-A) is associated with the expression of IL-10^20^. We then evaluated the levels of NFI-A in the Mos after exposing to miR-exosomes. Indeed, the exposure to the miR-exosomes increased the levels of NFI-A in the Mos (Fig. 2E). We then knocked down the NFI-A gene in Mos (Fig. 2F), and exposed the NFI-A-null Mos to miR-exosomes in the culture; the expression of IL-10 was abolished (Fig. 2A–D). Since NF-κB is associated with a number of immune responses, we wondered if NF-κB was necessary for the miR-146a-induced IL-10 expression in Mos. To this end, we knocked down the NF-κB gene in Mos, then exposed the NF-κB-null Mos to miR-exosomes in the culture. As analyzed by qPCR and Western blotting, no detectable effect of the NF-κB-deficiency on the induction of IL-10 in Mos was observed (Fig. S4).

It is reported that STAT3 is involved in the IL-10 expression^21^. We infer that STAT3 may be associated with the miR-146a-induced IL-10 expression. To test the inference, we analyzed the STAT3 phosphorylation in Mos after exposure to the miR-exam. The results showed that levels of phosphorylated STAT3 markedly increased after exposure to miR-exosomes in the culture (Fig. 2G). The results suggest that STAT3 may be associated with the increase in NFI-A expression in Mos induced by miR-146a. To strengthen the data, in separate experiments, we added a STAT3 inhibitor to the culture of Mos in the presence of miR-exosomes. The increase in NFI-A expression was inhibited (Fig. 2G).

### miR-146a-conditioned Mos show immune suppressor ability

**Figure 3:**
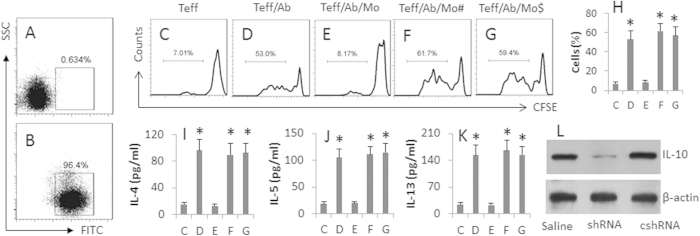
miR-146a-conditioned Mos suppress Teff cell activities. (**A**,**B**) naïve Mos were cultured in the presence of FITC-labeled miR-exosomes for 6 h and analyzed by flow cytometry. The dot plots indicate the naïve control Mos (**A**) and the miR-146a-exosome-laden Mos (**B**). (**C**–**G**) miR-146a-conditioned Mos were prepared. CD4^+^ CD25^−^ T effector (Teff) cells were isolated from PBMC of healthy subjects and labeled with CFSE. The experimental procedures were denoted above each histogram. The flow cytometry histograms indicate the frequency of Teff cell proliferation. (**H**) the bars indicate the summarized data of (**C**–**G**). (**I**–**K**) the bars indicate the Th2 cytokines in the culture supernatant (assessed by ELISA). Mo: Mos were conditioned with the miR-exosomes. #Mos were isolated from the spleen of the IL-10-deficient mice. $: Naïve Mos. L, the conditioned Mos were treated with saline, or shRNA of IL-10 (shRNA), or control shRNA (cshRNA). The Western blots show the IL-10 gene knockdown results. The data of bars are presented as mean ± SD. *p < 0.01, compared with group (**C**) The data are a representative of 3 independent experiments.

Based on the fact that IL-10-producing Tregs and Bregs have immune suppressor functions, we hypothesize that the miR-146a-conditioned Mos (IL-10 positive) are capable of suppressing immune effector cell activities. To test the hypothesis, we conditioned naïve Mos with the miR-exosomes. The flow cytometry data showed that more than 95% Mos took the miR-exosomes in the culture (Fig. 3A,B). The Mos were cultured with CD4^+^ CD25^−^ effector T (Teff) cells in the presence of anti-CD3/CD28 antibodies. As evaluated by flow cytometry, the presence of Mos markedly suppressed the Teff cellular activities, including T cell proliferation (Fig. 3C–H) and release of Th2 cytokines (Fig. 3I–K). To test if IL-10 was the mediator by which the Mos suppressed the Teff cell activities, in separate experiments, the Mos were replaced with IL-10-null Mos (isolated from the spleen of IL-10-deficient mice); the suppressor ability of the Mos was abolished (Fig. 3F,H–K). On the other hand, the presence of naïve Mos did not affect the Teff cell activities (Fig. 3G–K). The results demonstrate that the miR-146a-conditioned Mos have the properties of immune suppressor cells.

### Adoptive transfer with IL-10-producing Mos or the nasal drop of miR-exosomes suppresses Th2 polarization in an AR mouse model

**Figure 4:**
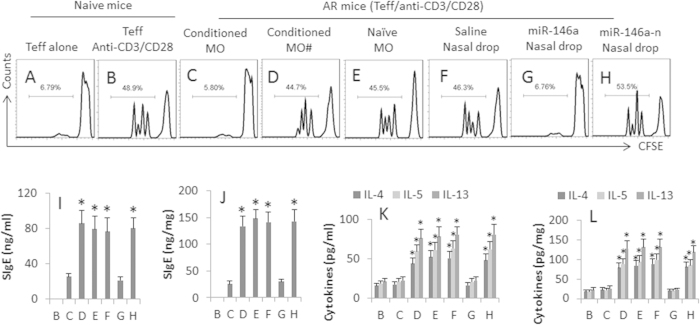
Adoptive transfer of IL-10-producing Mos suppresses skewed Th2 polarization in mice with AR. A mouse AR model was created in BALB/c mice with OVA as the specific antigen. The AR mice were adoptively transferred with conditioned (or naïve) Mos (10^6^ cells/mouse, via tail vein injection), or nasal drops containing miR-exosomes (10 μg/ml; 10 μl/nostril, 3 times/day), or nasal drops containing miR-146a-n (n = null) exosomes. The treatments are denoted above each subpanel of (**A**–**G**) Spleen cells, serum and specimens of nasal mucosal extracts were prepared and analyzed by flow cytometry and ELISA. (**A**–**G**) the flow cytometry histograms indicate the frequency of the Teff proliferation. (**I**,**J**) the bars indicate the levels of OVA-specific IgE in the serum (**I**) and nasal mucosal extracts (**J**). (**K**,**L**) the bars indicate the Th2 cytokine levels in the serum (**J**) and nasal mucosal extracts (**L**). #Mos were isolated from the spleen of the IL-10-deficient mice. miR-146a-nasal drop: AR mice were treated with nasal drops containing the miR-146a-exosomes. *p < 0.01, compared with group A (**H**), or group B (**I**–**L**). Each group consists of 6 mice. The data are a representative of 6 independent experiments.

Following published procedures^7^ with a minor modification, we created a mouse AR model with the OVA as a specific antigen. A group of AR mice was adoptively transferred with conditioned Mos (Mos were exposed to miR-exosomes in the culture) on day 1 and day 7 respectively. After sacrifice, we collected the spleen, serum and nasal mucosa from the mice; the specimens were analyzed by flow cytometry and ELISA. The results showed that an OVA-specific spleen Teff cell proliferation was induced in the culture from AR mice, which was markedly suppressed in mice received the conditioned Mos, or nasal drops containing the miR-exosomes (Fig. 4A–H).

OVA-specific IgE was detected in the serum and nasal mucosal extracts of AR mice, but not in the saline control mice (Fig. 4I,J). Low levels of IL-4, IL-5 and IL-13 were detected in the serum and nasal mucosal extracts of naive control mice, which was significantly higher in AR mice (Fig. 4K,L). In the AR mice, adoptively transferred with the conditioned Mos, the levels of OVA-specific IgE, IL-4, IL-5 and IL-13 in the serum and nasal mucosal extracts were much lower than the AR mice treated with saline. Adoptive transfer with naïve Mos did not alter the Th2 polarization in the AR mice (Fig. 4K,L). To strengthen the results, a group of AR mice was treated with a nasal drop containing the miR-exosomes, which dramatically suppressed the skewed Th2 polarization in the AR mice (Fig. 4F–L). The results indicate that the adoptive transfer with the conditioned Mos, or administration of a nasal drop containing miR-exosomes, can suppress the skewed Th2 polarization in AR mice.

**Figure 5:**
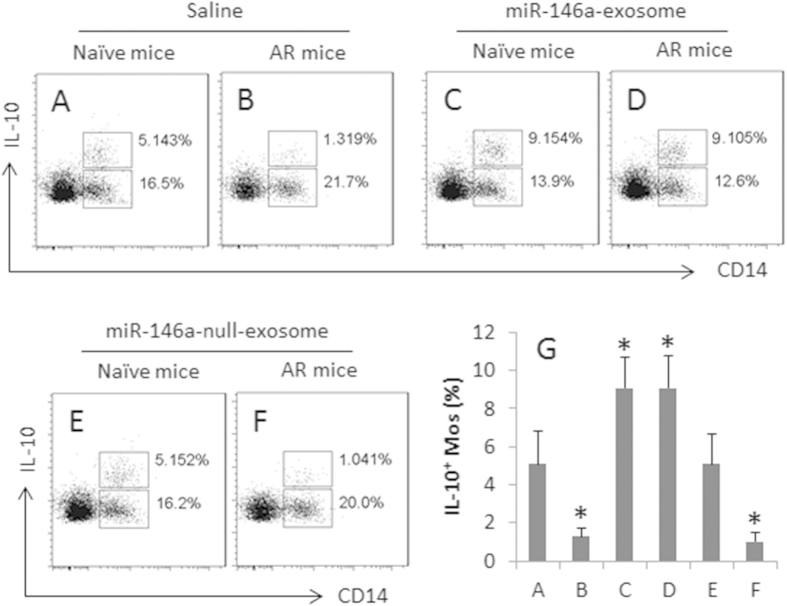
MiR-146a induces IL-10^+^ Mos in the nasal mucosa. Naïve mice and AR mice were treated with a nasal drop containing saline or miR-exosomes (10 μg/ml saline; 10 μl/nostril; 3 times a day) for 1 week. The whole nasal mucosa was excised; single cells were made by enzymatic digestion. The cells were analyzed by flow cytometry. (**A**–**F**) the dot plots of the upper gates indicate the frequency of IL-10^+^ Mos. The lower gates show the frequency of IL-10^−^ Mos. (**G**) the bars indicate the summarized data (mean ± SD) of the IL-10^+^ Mos in (**A**–**F**) *p < 0.01, compared with group A. Each group consists of 9 mice. Cells isolated from 3 mice were pooled as one sample. The data are a representative of 3 independent experiments.

To elucidate if the miR-146a induced IL-10-producing Mos in the nasal mucosa, in separate experiments, naïve mice and AR mice were treated with saline or a miR-exosomes-containing nasal drop for one week. As shown by flow cytometry data, the treatment with miR-exosomes markedly increased the frequency of IL-10^+^ Mos in the nasal mucosa in both naive mice and AR mice (Fig. 5A–D,G), which did not occur when the mice treated with the nasal drops containing the miR-146a-null-exosomes (Fig. 5E–G).

## Discussion

Although it is well known that the skewed Th2 polarization plays a critical role in the pathogenesis of allergic diseases, such a pathological condition is refractory to be corrected. The present study has shown that the epithelial cell-derived miR-146a can induce the expression of IL-10 in Mos. The IL-10-producing Mos are capable of suppressing other Teff cell activities and inhibiting the Th2 polarization in the nasal mucosa of a mouse AR model.

It is well known that dendritic cells (DC) and macrophages are important in the immunity. In the aspect of immune regulation, the tolerogenic DCs produce TGF-β or other immune regulatory molecules to induce Tregs; the latter suppresses skewed immune responses, such as Chen *et al.* indicate that the intestinal epithelial cell-derived αvβ6 induces naïve DCs to be tolerogenic DCs, which further induce Tregs in the intestine^22^. Besides the role in the innate immunity, macrophages are also important in the immune regulation. A fraction of macrophages can be activated by Th2 cytokines and designated as the alternatively activated macrophages^23^. In fact, both DCs and macrophages are differentiated from Mos. The present data show that Mos can be modulated to be immune regulatory cells. The CD14^+^ CD16^−^ monocytes are also called the classic Mos, which have the potential to differentiate into DCs or macrophages. We observed that the major compatibility complex II (MHC II) was below detectable levels the IL-10-producing Mos (data not shown), indicating that the IL-10-producing Mos are neither DCs nor macrophages. In other words, the classic Mos may differentiate into DCs, or macrophages, or immune regulatory cells, depending on the cytokine environment. Prasse *et al.* indicate that the IL-10-producing Mos may further develop into the alternative activated macrophages^24^.

The present data show that the epithelial cell-derived miR-146a can induce the expression of IL-10 in CD14^+^ CD16^−^ Mos. IL-10 is an immune suppressive cytokine; it can be generated by a number of cells. The IL-10-producing DCs, T cells and B cells have immune suppressive functions, which can be induced by exposure to extrinsic IL-10, or activation of Toll like receptors, such as LPS can induce IL-10 production by DCs^25^ or B cells^26^; these cells thus obtain the immune suppressive ability^25^,^26^. Our data are consistent with these previous studies by showing that the IL-10-producing Mos also have the immune regulatory ability.

The present data show a functional facet of miR-146a that contributes to immune regulation by inducing a fraction of regulatory Mos. Our data are consistent with previous studies. Lu *et al.* indicate that miR-146a is required in maintaining the functional homeostasis of Tregs; deficiency of miR-146a results in fatal IFNγ-dependent immune-mediated lesions in a variety of organs^27^. Taganov *et al.* indicate that miR-146 in control of Toll-like receptor and cytokine signaling through a negative feedback regulation loop involving down-regulation of IL-1 receptor-associated kinase 1 and TNF receptor-associated factor 6 protein levels^16^. Others observed that impaired miR-146a expression links subclinical inflammation and insulin resistance in Type 2 diabetes^28^.

In summary, the present data indicate that miR-146a can induce IL-10-producing Mos to suppress the skewed Th2 polarization, suggesting that miR-146a has the potential in the treatment of allergic disorders, such as allergic rhinitis.

## Materials and Methods

### Reagents

The antibody of IL-10, ELISA kits of IL-4, IL-5 and IL-13 were purchased from R&D Systems (Shanghai, China). The anti-LMP1 antibody was purchased from Santa Cruz Biotech (Shanghai, China). The OVA-specific IgE ELISA kit was purchased from AbD Serotec (Shenzhen, China). MicroRNA Reverse Transcription kit and TaqMan miR assay primers for human miR-146a were provided by Applied Biosystems (Shanghai, China). The FITC-labeling kit and LPS were purchased from Sigma Aldrich (Shanghai, China). Reagents for RT-qPCR, Western blotting and the miR transfection were purchased from Invitrogen (Shanghai, China). Immune cell isolation kits were purchased from Miltenyi Biotech (Shanghai, China). Before experiments, all reagents and solutions were checked by the Limulus amebocyte lysate test to get rid of the possibility of endotoxin contamination. The human-miR-146a locked nucleic acid (LNA) and control LNA were purchased from Ribotask (Shanghai, China). The miR-146a, antisense of miR-146a and IL-10 promoter reporter gene construct were provided by Genescript (Nanjing, China).

### Ethic statement

The using human tissue and mice in the present study was approved by the Human or Animal Research Ethic Committee at Shanghai Jiaotong University; all the procedures were carried out in accordance with the guidelines. An informed, written consent was obtained from each subject.

### Collection of human nasal epithelial specimens

Using a plastic curette, nasal epithelial specimens were scraped from the surface of the inferior nasal turbinates of patients with allergic rhinitis (AR) and healthy subjects. The diagnosis of AR was carried out by Otolaryngologists in our department based on the criterion of a typical history of allergic rhinitis more than 2 years; running nose, positive skin prick test against specific antigens, serum specific IgE levels were greater than 0.3 IU/ml. Subjects with sinusitis were excluded.

### Mice

Male BALB/c mice and IL-10-deficient mice (BALB/c background) were purchased from the Xinmao Experimental Animal Institute (Shanghai, China). The mice were maintained in a pathogen-free environment with accessing to food and water freely.

### Extraction of RNA and proteins

The total RNA and proteins were extracted from the specimens following the routine procedures in our laboratory^29^. The cDNA was synthesized using a reverse transcription kit following the manufacturer’s instructions. The mirVana™ miRNA Isolation Kit (Life Technology) was used for RNA isolation of miR detection.

### Real time quantitative PCR (qPCR)

The qPCR was carried out in a qPCR device (MiniOpticon Real Time PCR System, Bio-Rad). The cDNA was synthesized using a TaqMan MicroRNA Reverse Transcription kit with TaqMan miR assay primers for human miR-146a. The results were calculated with the 2^−ΔΔCt^ method and normalized to a percentage of the internal control gene of β-actin for IL-10; levels of miR-146a were normalized to RNA U6 controls. Primers of IL-10 include: Forward, gttctttggggagccaacag; reverse, gctccctggtttctcttcct. The primers of β-actin include: Forward, cgcaaagacctgtatgccaa; reverse, cacacagagtacttgcgctc.

### Western blotting

The proteins (50 μg/well) were fractioned by SDS-PAGE (sodium dodecyl sulfate polyacrylamide gel electrophoresis) and transferred onto a PVDF membrane. After blocking with 5% skim milk for 30 min, the membrane was incubated with the primary antibodies (200 ng/ml) for 1 h at room temperature and followed by incubating with the second antibodies (conjugated with horseradish peroxidase) for 1 h. The membrane was washed with TBST (Tris-buffered saline-Tween 20) after each incubation. The blots on the membrane were developed with ECL (enhanced luminol-based chemiluminescent substrate). The results were photographed with a KODAK Image Station 4000 mm Pro (KODAK, Shanghai, China). The integrated density of the blots was determined by PhotoShop Software (CS5).

### Cell culture

The RPMI2650 cells (a human airway epithelial cell line; ATCC, USA) and A549 cells (a mouse airway epithelial cell line; ATCC, USA) were cultured in DMEM (Dulbecco's Modified Eagle Medium) supplemented with 10 fetal bovine serum, 100 U/ml penicillin, 0.1 mg/ml streptomycin and 2 mM L-glutamine. As checked with the Trypan blue exclusion assay, the cell viability was greater than 98%.

### Purification of miR-146a-exosomes (miR-exosomes)

RPMI2650 cells or A549 cells were cultured in a 75 cm^2^ flask. When the cells reached confluence (about 10^7^ cells/ml), LPS was added to the culture at a concentration of 100 pg/ml; the culture was continued overnight. Exosomes were purified from the culture supernatant following our established procedures^30^. Briefly, supernatant was harvested and centrifuged at 300 × g (10 min), 1200 × g (20 min), and 10,000 × g (30 min) to remove cell debris. Exosomes were pelleted at 100,000 × g for 1 h and resuspended in PBS for subsequent studies. The miR-146a in the exosomes were quantified by qPCR first, and then converted to miR-146a/ml exosome in order to be used in the experiments. About 10–20 μg miR-146a was harvested in the supernatant (20 ml) collected from one 75 cm^2^ flask as measured by qPCR. To generate the miR-146a-null exosomes (con-exosomes), A549 cells were transfected with a locked nucleic acid (LNA)-modified antisense oligonucleotides of miR-146a (an antisense of miR-146a; 1 μg/10^6^ cells), or a control LNA, with the aid of electroporation (Mocroporator, Digital Bio; 1200 V, 20 ms, 1 pulse) using the Neon Transfection system following the manufacturer’s instructions. The miR-146a-null A549 cells were then used to generate the miR-146a-null exosomes in the same procedures above. A portion of exosomes were labeled with FITC with a FITC-labeling kit following the manufacturer’s instructions.

### Enzyme-linked immunosorbent assay (ELISA)

Cytokine levels were determined by ELISA with the commercial reagent kits following the manufacturer’s instructions.

### Methylation specific PCR

Genomic DNA was isolated using TRIzol reagent following the manufacturer’s instructions. The DNA was modified by bisulfite treatment for 12 h using a DNA Methylation kit (Sigma Aldrich) following the manufacturer’s instructions. This treatment deaminates unmethylated cytosines into uracil but does not affect 5-methylcytosines. Following bisulfite treatment, PCR amplification was performed. DNA samples were then purified with the PureLink® Kits (Life Techology) according to the manufacturer's recommendations. The amplified fragments were assessed for the methylation status by qPCR with primers specific to methylated and unmethylated templates, respectively. The results were also normalized to a percentage of the input DNA. The primers of IL-10 promoter using in this experiment are include: Methylated, forward: tttggaatatattttgtgatttcgt; reverse: tcaactataaattctcattcgcgta. Unmethylated, forward: tttggaatatattttgtgattttgt; reverse: ccctcaactataaattctcattcaca.

### Immune cell isolation

The immune cells were isolated by the magnetic cell sorting (MACS) with commercial reagent kits following the manufacturer’s instructions. The purity of the isolated cells was greater than 98% as assessed by flow cytometry.

### Mos were conditioned with miR-exosomes

CD14^+^ CD16^−^ Mos were isolated from PBMCs with MACS. The Mos were cultured in RPMI1640 medium in the presence of miR-146a (10 μg/ml), PMA (20 ng/ml) and IL-7 (20 ng/ml) for 6 days. As checked by flow cytometry, the frequency of IL-10^+^ Mos was greater than 98%.

### Assessment of effector T cell (Teff cell) proliferation

The blood was obtained from healthy subjects. The peripheral blood mononuclear cells (PBMC) were isolated from the blood by gradient density centrifugation. The naïve CD4^+^ Teff cells were isolated from the PBMC by MACS following the manufacturer’s instructions and labeled with CFSE (Carboxyfluorescein succinimidyl ester). The Teff cells were cultured with the IL-10^+^ Mos at a ratio of 1: 1 for 3 days in the presence of PMA (20 ng/ml). The cells were collected and analyzed by flow cytometry (the CFSE-dilution assay).

### Nasal allergy mouse model development

The using mice in the present study were approved by the Animal Ethic Committee at Shenzhen University; the procedures are in accordance to the guidelines. BALB/c mice were purchased from Guangdong Experimental Animal Center. Mice were intrapenitoneally injected with 100 μl of PBS containing 100 μg of OVA and 1 μg of cholera toxin on day 0 and day 7. Nasal antigen challenges were performed on days 15, 16 and 19 with 100 μg of OVA as previously described^(7)^ with a minor modification. The mice were sacrificed next day of the last nasal challenge. Samples of the blood, spleen and nasal mucosa were collected for further experiments.

### Isolation of mononuclear cells from nasal mucosa

The nasal mucosa was collected from the whole nasal cavity of mice and incubated in culture medium containing 5mM EDTA (Ethylenediaminetetraaceticacid) for 1 at 37 °C. The mucosa was then cut into small pieces and incubated in culture medium containing collagenase IV (0.5 mg/ml) for 1 h at 37 °C. The tissue slurry was passed through a cell strainer (70 μm in diameter) to remove the un-digested tissue, and then the mononuclear cells were further isolated by percoll density gradient centrifugation. The cell viability was greater than 96% as assessed by Trypan blue exclusion assay. The cells were cultured in RPMI1640 medium for further experiments.

### Flow cytometry

Cells were fixed with 2% paraformaldehyde for 2 h at room temperature. The cells were then incubated with 0.5% saponin for 30 min to increase the permeability of the cell membrane. After washing with phosphate buffered saline, the cells were incubated with fluorochrome-labeled antibodies for 1 h. The stained cells were analyzed with a flow cytometer (FACSCanto II; BD Biosciences).

### IL-10 gene silencing in Mos

Naive Mos were isolated from PBMC and treated with commercial reagent kits of shRNA of IL-10 or control shRNA following the manufacturer’s instructions. The Mos were then treated with miR-exosomes (10 μg/ml) in the culture for 6 days (in order to increase the expression of IL-10 in the Mos). The gene knockdown effect was assessed by Western blotting.

### Transduction of IL-10 promoter luciferase reporter

The luciferase reporter constructs were provided by Genescript (Nanjing, China) with the IL-10 promoter sequence (-966 to -1) linked to a luciferase gene. The sequences were cloned into an adenovirus vector. The control vector did not contain the IL-10 promoter sequence. The reporter vectors were transduced into Mos; the Mos were then exposed to miR-exosomes or con-exosomes in the culture following the manufacturer’s instruction. Luciferase activity in cell lysates was determined using a Sirius model luminometer (Berthold Technologies).

### Statistics

The data are presented as mean ± SD. The differences between groups were determined by Student t test, or ANOVA if more than two groups. A p < 0.05 was set as a significant criterion.

## Additional Information

**How to cite this article**: Luo, X. *et al.* Epithelial cell-derived micro RNA-146a generates interleukin-10-producing monocytes to inhibit nasal allergy. *Sci. Rep.* **5**, 15937; doi: 10.1038/srep15937 (2015).

## Supplementary Material

Supplementary Information
