# Supplementary material for: Epithelial cell-derived micro RNA-146a generates interleukin-10-producing monocytes to inhibit nasal allergy
Source: Sci Rep. 2015 Nov 3;5:15937. doi: 10.1038/srep15937 (PMC4630644; doi:10.1038/srep15937)
Supplement: Supplementary Information [file srep15937-s1.pdf]

Supplemental materials

Epithelial cell-derived micro RNA-146a generates interleukin-10-producing monocytes to inhibit nasal allergy

Running title: miR-146a induces IL-10-producing monocytes

Xi Luo,[1,2,*] Miaomiao Han,[2,*] Jianqi Liu,[3,4,*] Yu Wang,[2] Xiangqian Luo,[1] Jing Zheng,[1] Shuai Wang[3], Zhigang Liu,[3] Dabo Liu,[1] Ping-Chang Yang,[3] Huabin Li,[1,2]

[1]Department of Otolaryngology, Guangzhou Women and Children's Medical Center, Guangzhou, China. [2]Department of Otolaryngology, Head and Neck Surgery, Xinhua Hospital, Shanghai Jiaotong University School of Medicine, Shanghai, China. [3]Allergy & Immunology Center, Shenzhen University School of Medicine, Shenzhen, China. [4]ENT Institute, Longgang Central Hospital, Shenzhen, China.

* These authors contributed equally to this study.

Corresponding authors: Dr. Dabo Liu, Dr. Huabin Li and Dr. Ping-Chang Yang (Department of Otolaryngology, Guangzhou Women and Children's Medical Center, Guangzhou, 510115, China. Email: daboliu@126.com. Department of Otolaryngology, Head and Neck Surgery, Xinhua Hospital, Shanghai Jiaotong University School of

Medicine, Shanghai, 200092, China. Email: allergyli@163.com. Allergy & Immunology Center, Shenzhen University School of Medicine, Shenzhen, 518060, China. Email: pcy2356@szu.edu.cn)

Supplemental figures

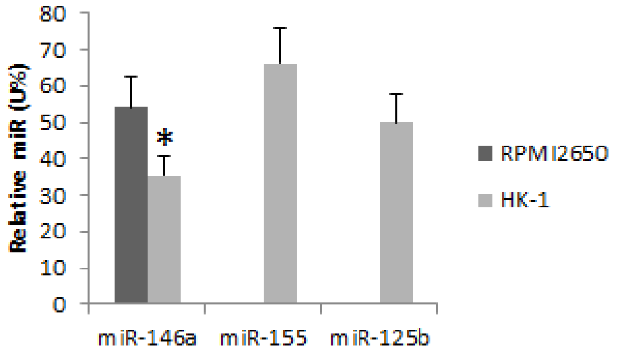

Figure S1. miR levels in RPMI2650 cell-derived exosomes and NPC cell-derived exosomes. RPMI2650 cells and HK-1 cells (a nasopharyngeal cancer cell line, using as a positive control) were cultured in the presence of LPS (100 ng/ml) for 48 h. Exosomes were purified from the culture supernatant. The total RNA was extracted from the exosomes. The levels of miR were determined by qPCR and presented as a relative value against U6. The data of bars are presented as mean ± SD. , p<0.01, compared with RPMI2650 cells. The data are representative of 3 independent experiments.

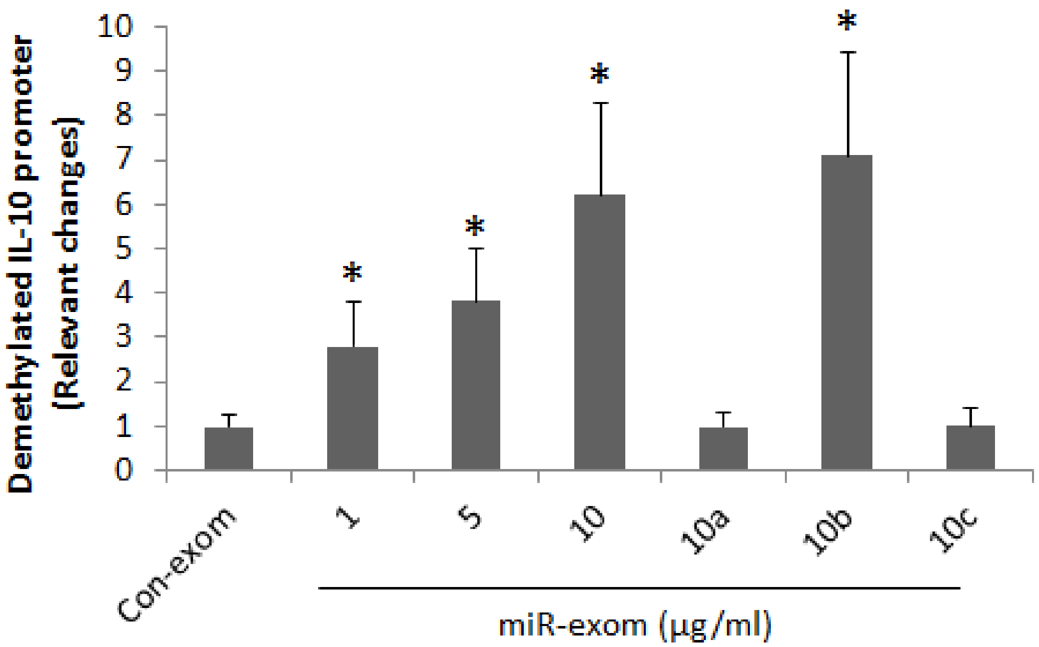

Figure S2. Demethylation of the IL-10 promoter. The $CD14^{+}$ $CD16^{-}$ Mos (Mo) were isolated from PBMC of healthy subjects and cultured for 6 days in the presence of miR-146a-exosomes at doses as denoted on the X axis and IL-7 (10 ng/ml). The bars indicate the demethylated DNA of the IL-10 promoter (assessed by specific methylation PCR). Con-exom: The miR-146a-null exosomes. miR-exom: The miR-146a-laden exosomes. 10a: The “a” indicates that NFI-A gene was knocked down in the Mos. 10b: The “b” indicates that Mos were treated with control shRNA. 10c, the “c” indicates the presence of antisense of miR-146a in the culture (10 μg/ml). The data of bars are presented as mean ± SD. *, p<0.01, compared with the con-exom group. The data are a representative of 3 independent experiments.

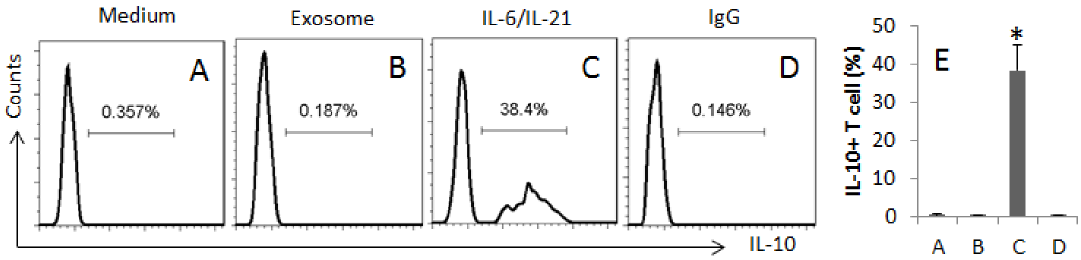

Figure S3. Induction of IL-10^+ CD4^+ T cells. CD4^+ CD25^- T cells were isolated from the peripheral blood of healthy subjects and cultured in the presence of exosomes (10 ng/ml) for 7 days, or (used as a positive control) following published procedures#, CD4^+ CD25^- T cells were placed in 24-well plate that were coated with anti-CD3 (1 µg/ml) and anti-CD28 (1 µg/ml) at 1×10^6 per well; cytokines were added in the culture, including IL-6 (20 ng/ml), IL-21 (50 ng/ml) and IL-2 (10 ng/ml) for 7 days. The cells were analyzed by flow cytometry. A-D, the histograms indicate the frequency of CD4^+ IL-10^+ T cells. E, the bars indicate the summarized data of A-D (mean ± SD; *, $p<0.01$, compared with group A). The data are representative of 3 independent experiments.

#, a reference: Jin JO, Han X, Yu Q. Interleukin-6 induces the generation of IL-10-producing Tr1 cells and suppresses autoimmune tissue inflammation. J Autoimmun. 2013 Feb; 40: 28-44.

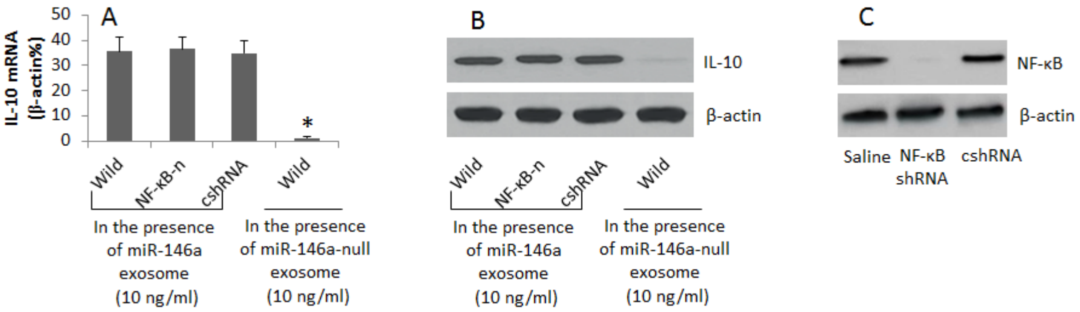

Figure S4. Assessment of the effect of NF-κB on the miR-146a-induced IL-10 in Mos. Naive Mos were isolated from the peripheral blood of healthy subjects by MACS. The wild or NF-κB-deficient (NF-κB-null) Mos were cultured in the presence of miR-146a-carrying exosomes or miR-146a-null (see Fig. 1E for detail) exosomes for 48 h. A, the bars indicate the IL-10 mRNA levels (mean ± SD; *, $p<0.01$, compared with the miR-146a-sufficient cells). B, the Western blots indicate the IL-10 protein levels. C, the results of NF-κB gene silence. The data are representative of 3 independent experiments.

Full length of Gel graphs (requested by the journal)

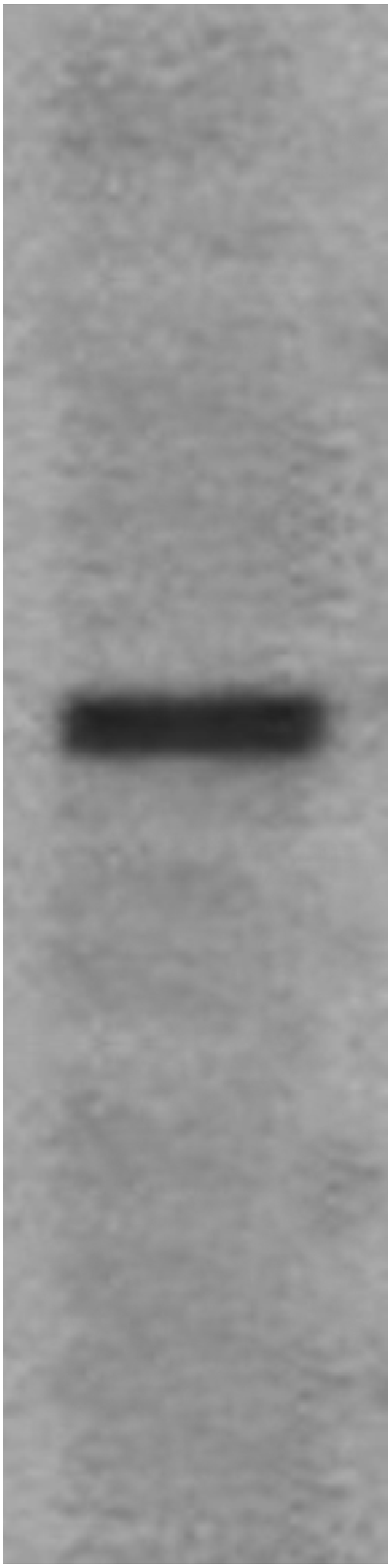

Figure 1D.

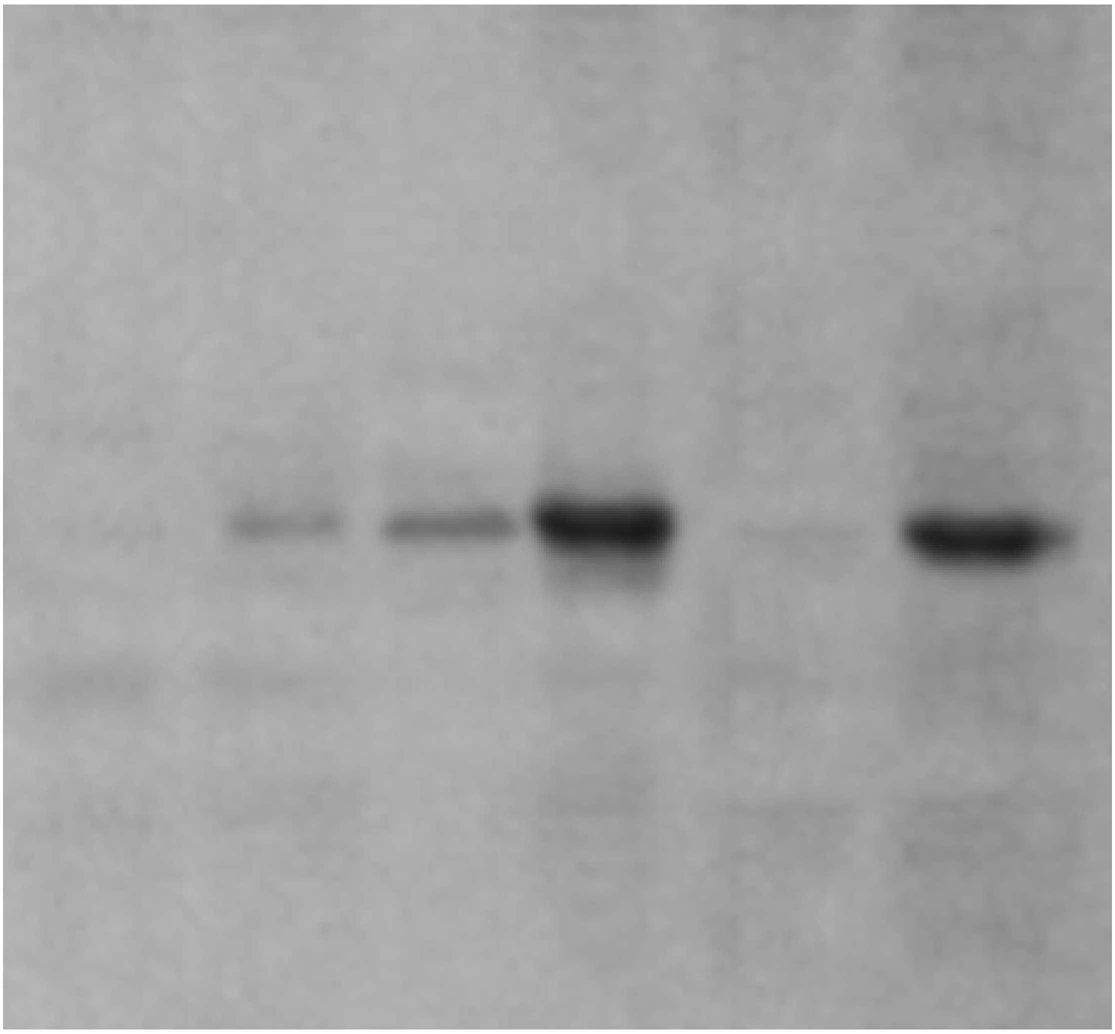

Fig. 2B. IL-10.

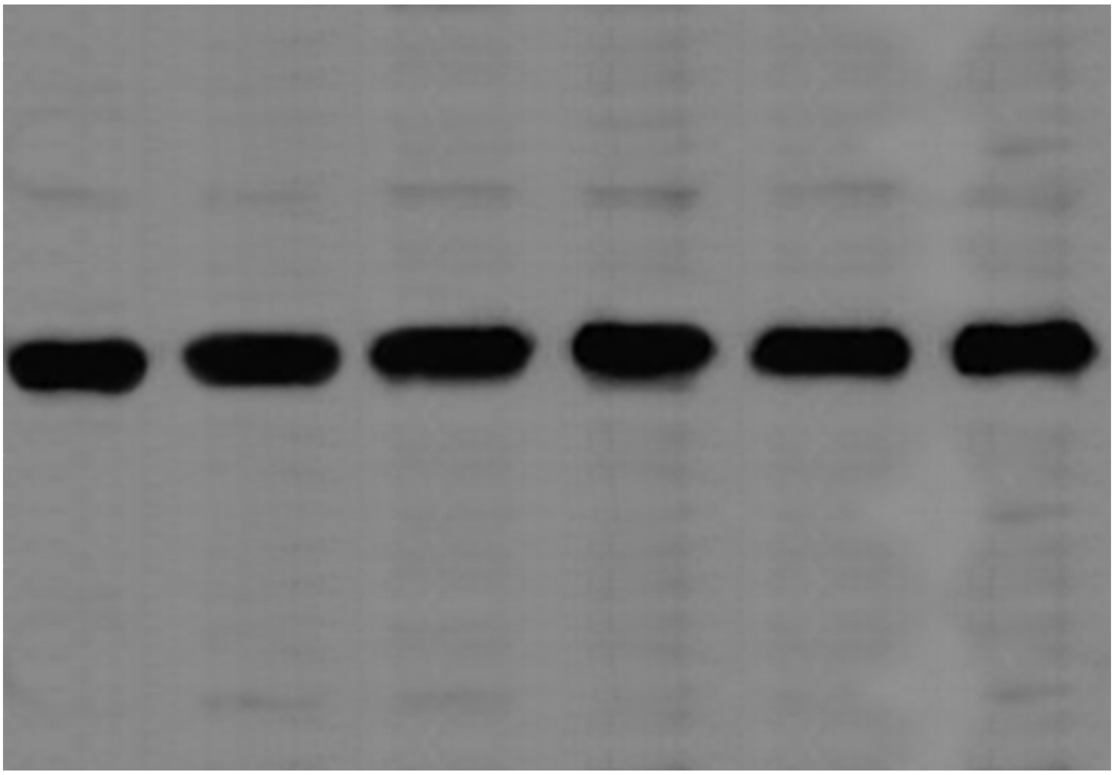

Figure 2B. Beta actin.

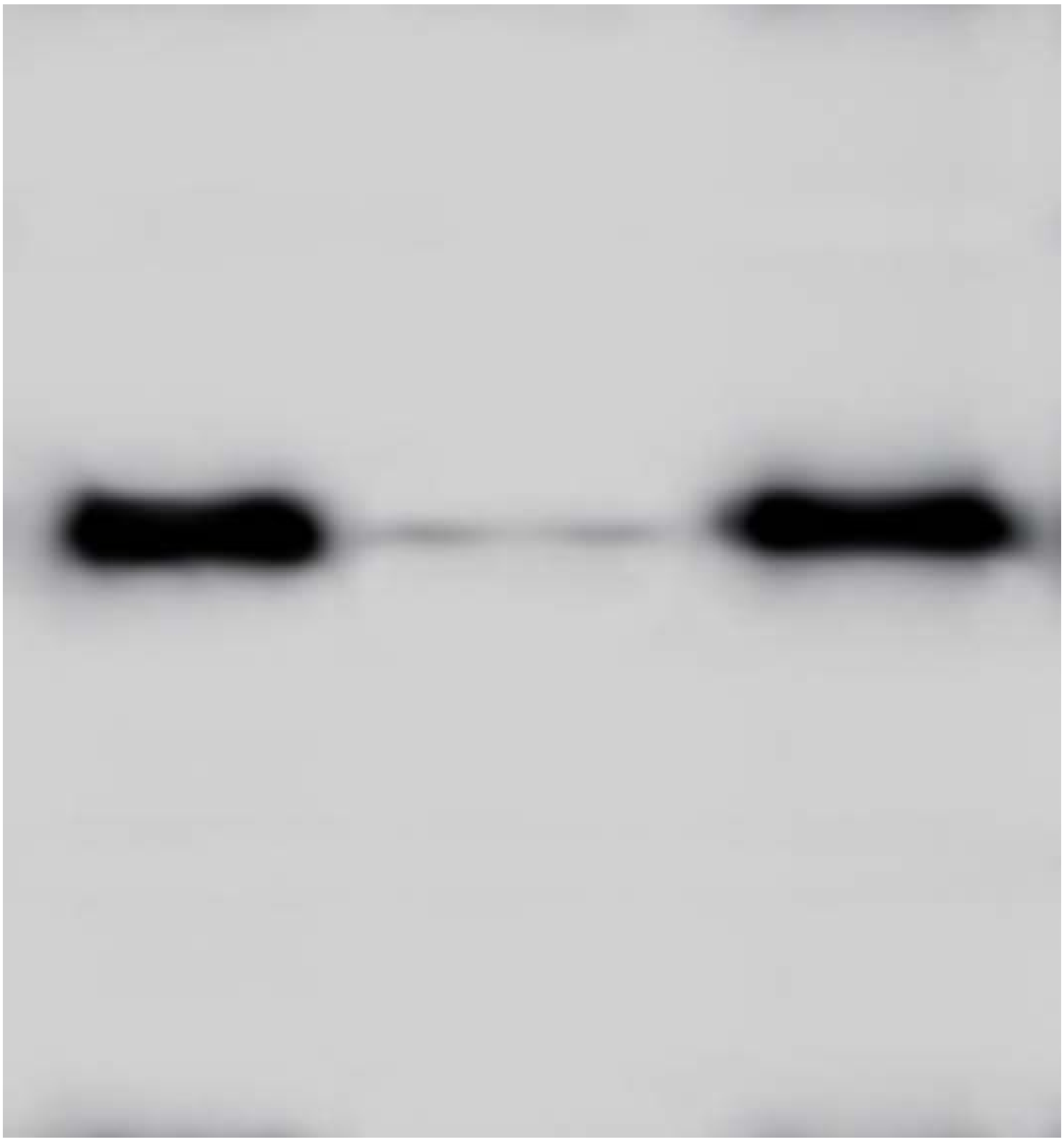

Figure 2E. NFI-A.

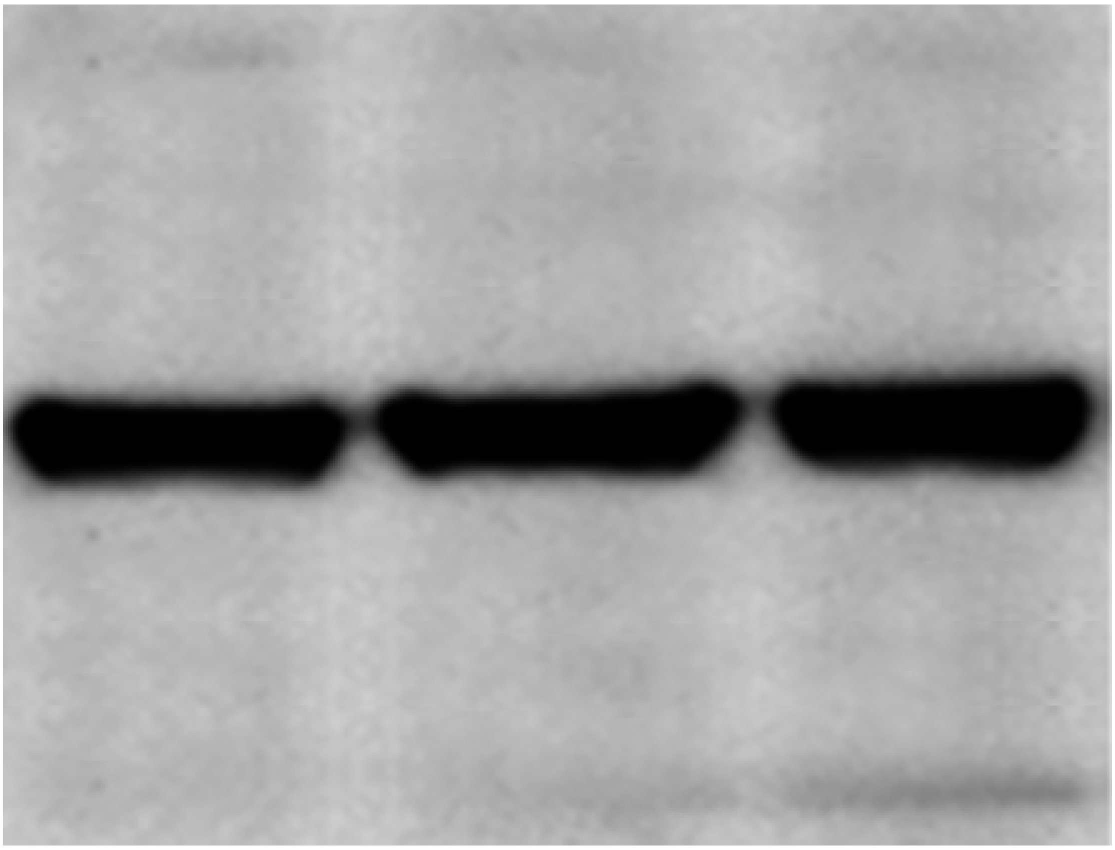

Figure 2E. Beta actin.

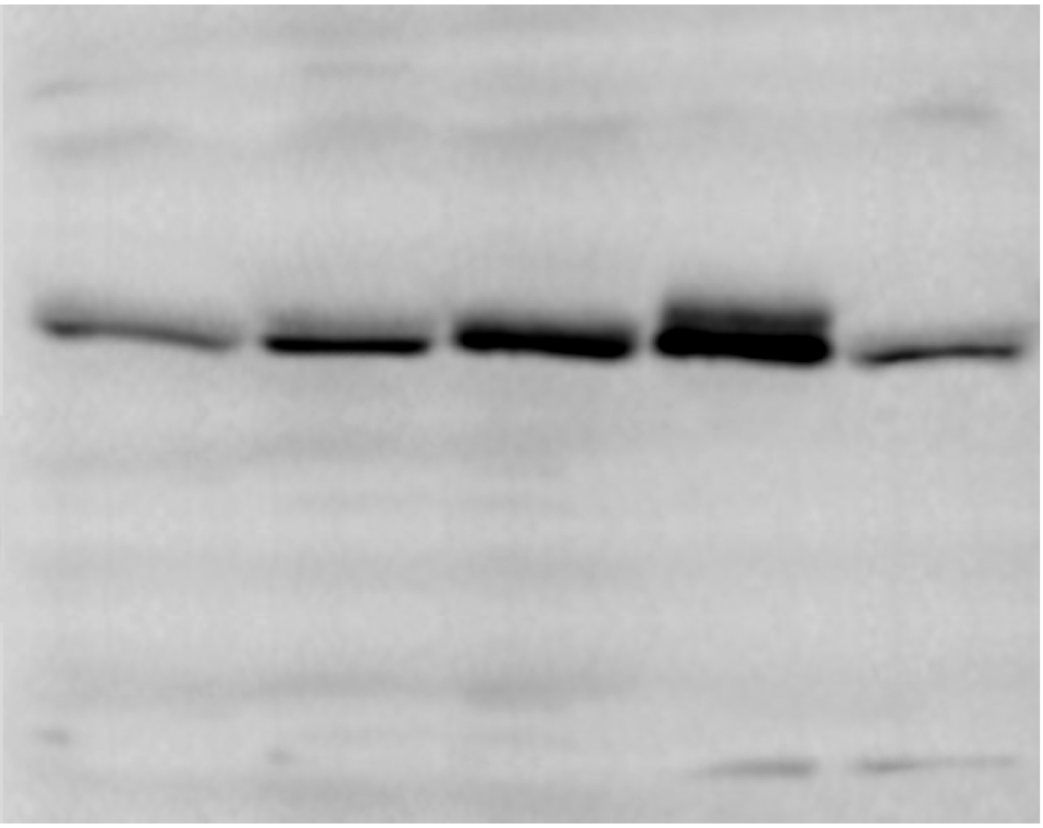

Figure 2F. NFI-A

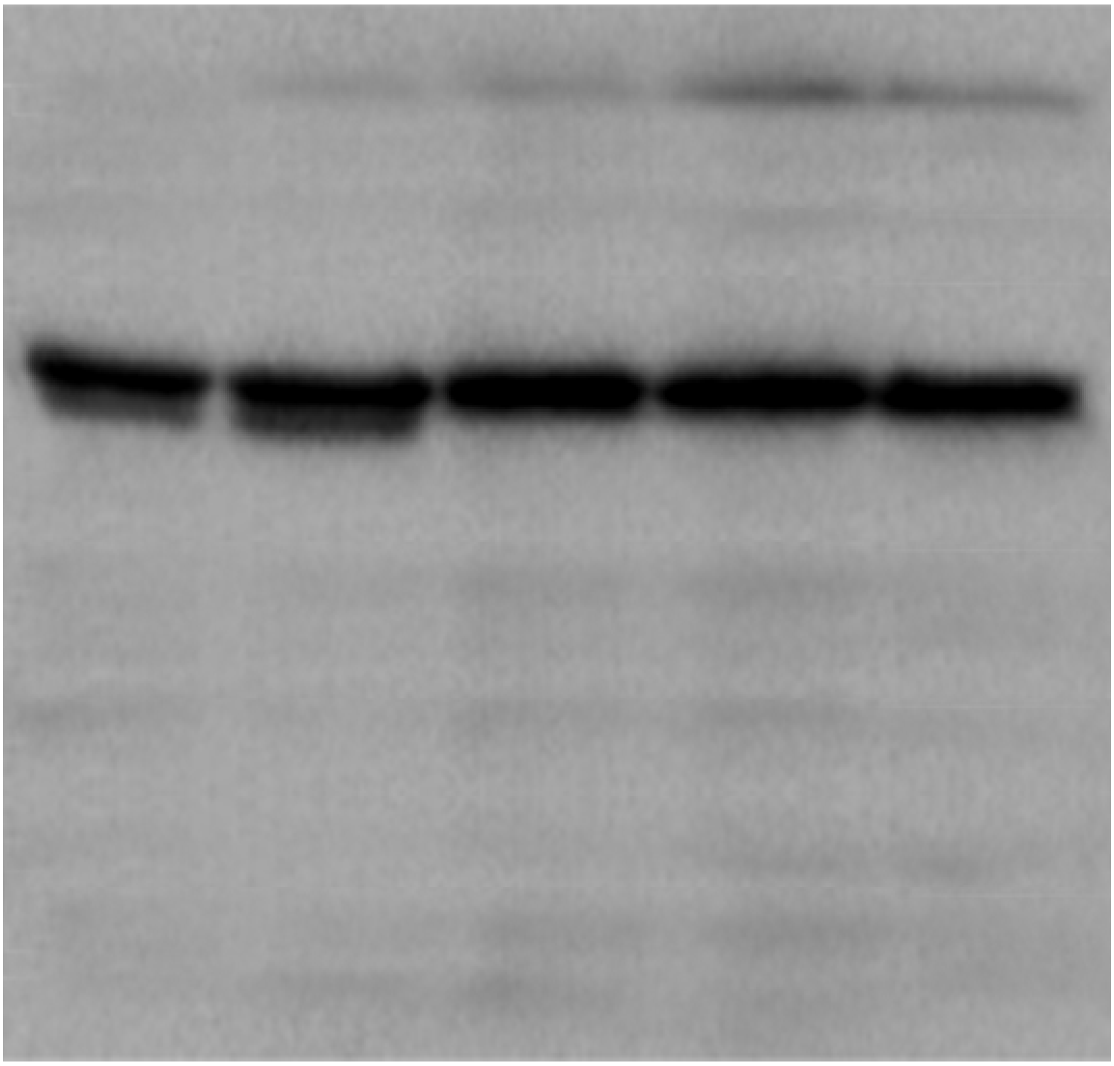

Figure 2F. Beta actin

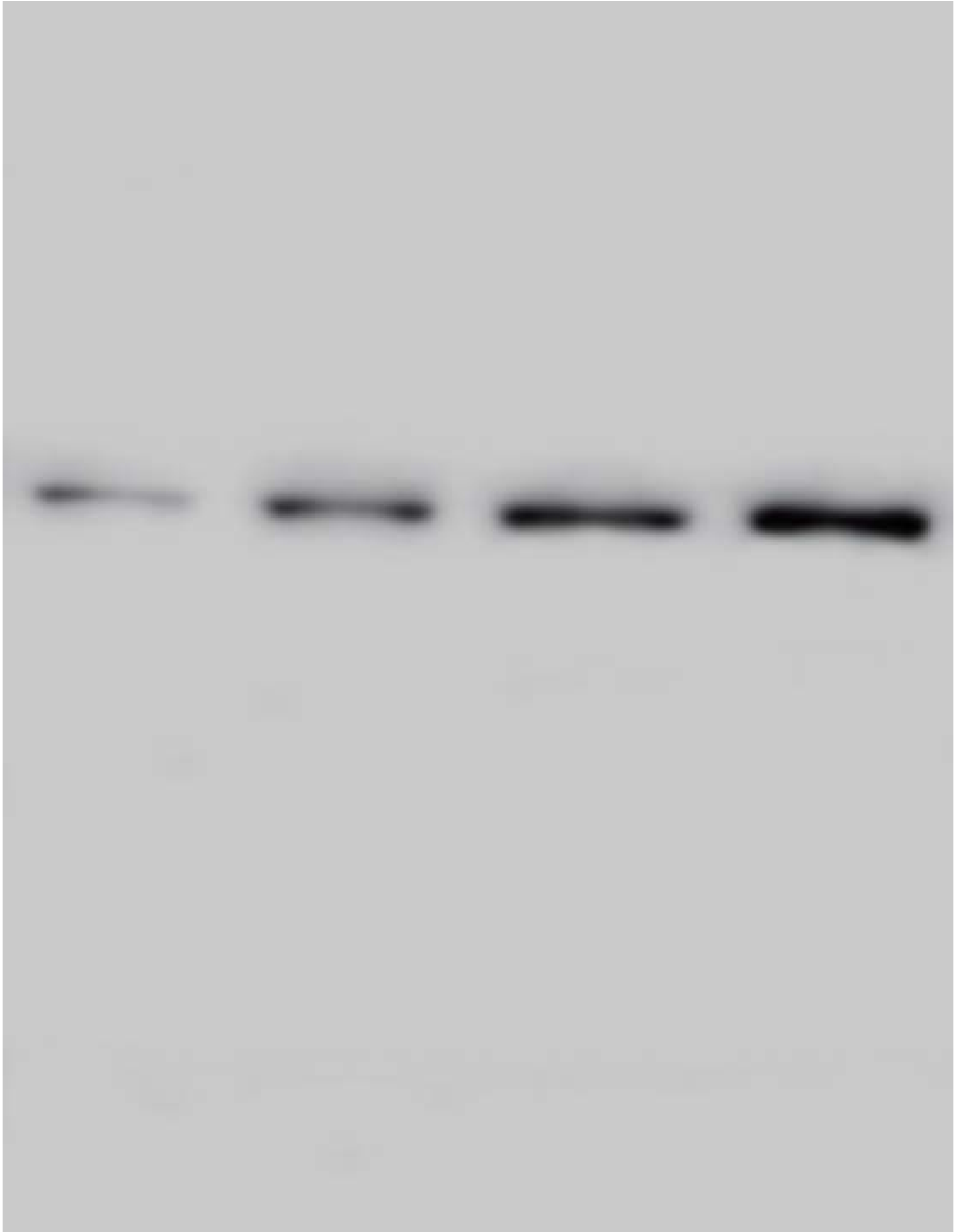

Figure 2G. STAT3-p

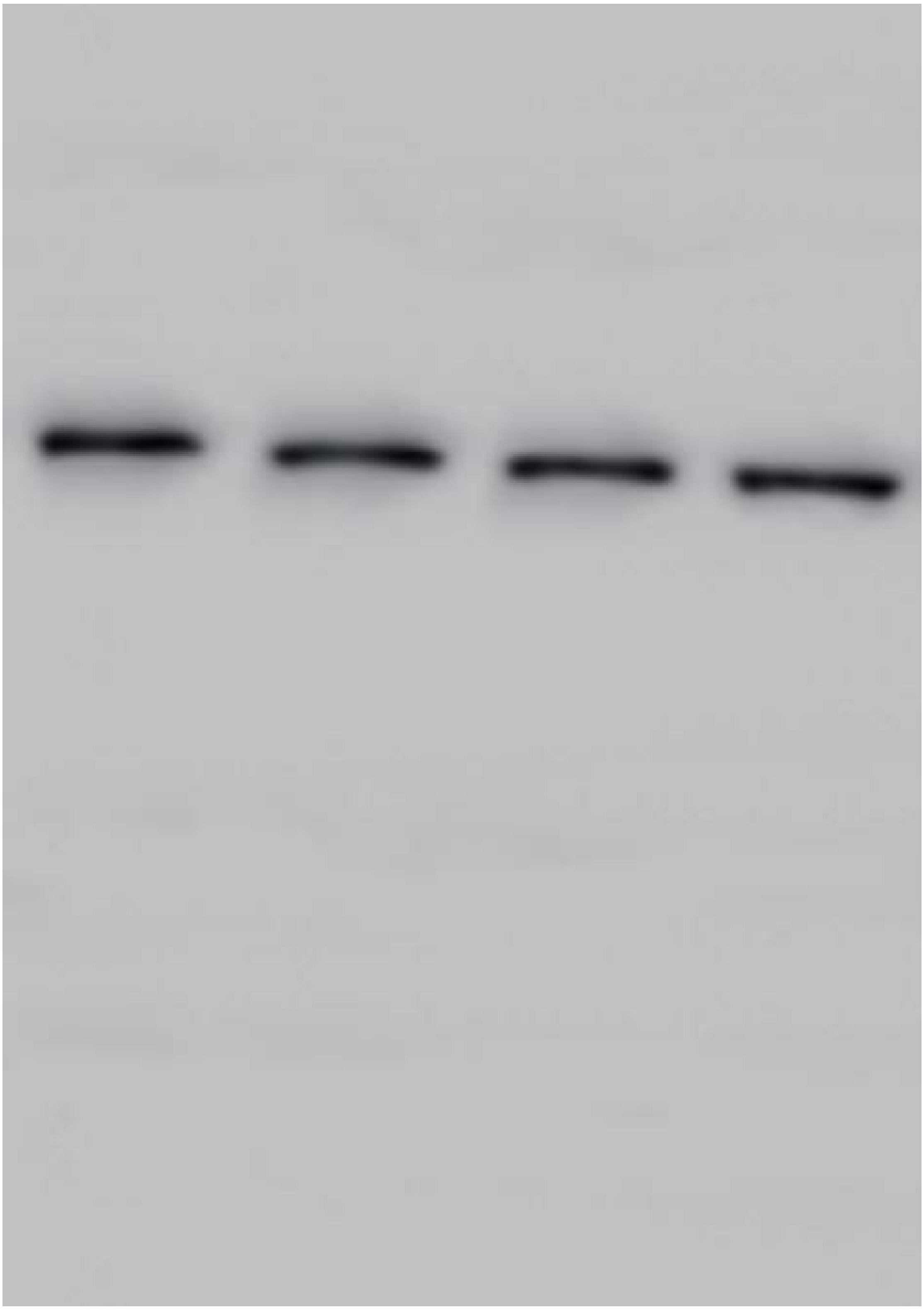

Figure 2G-STAT3

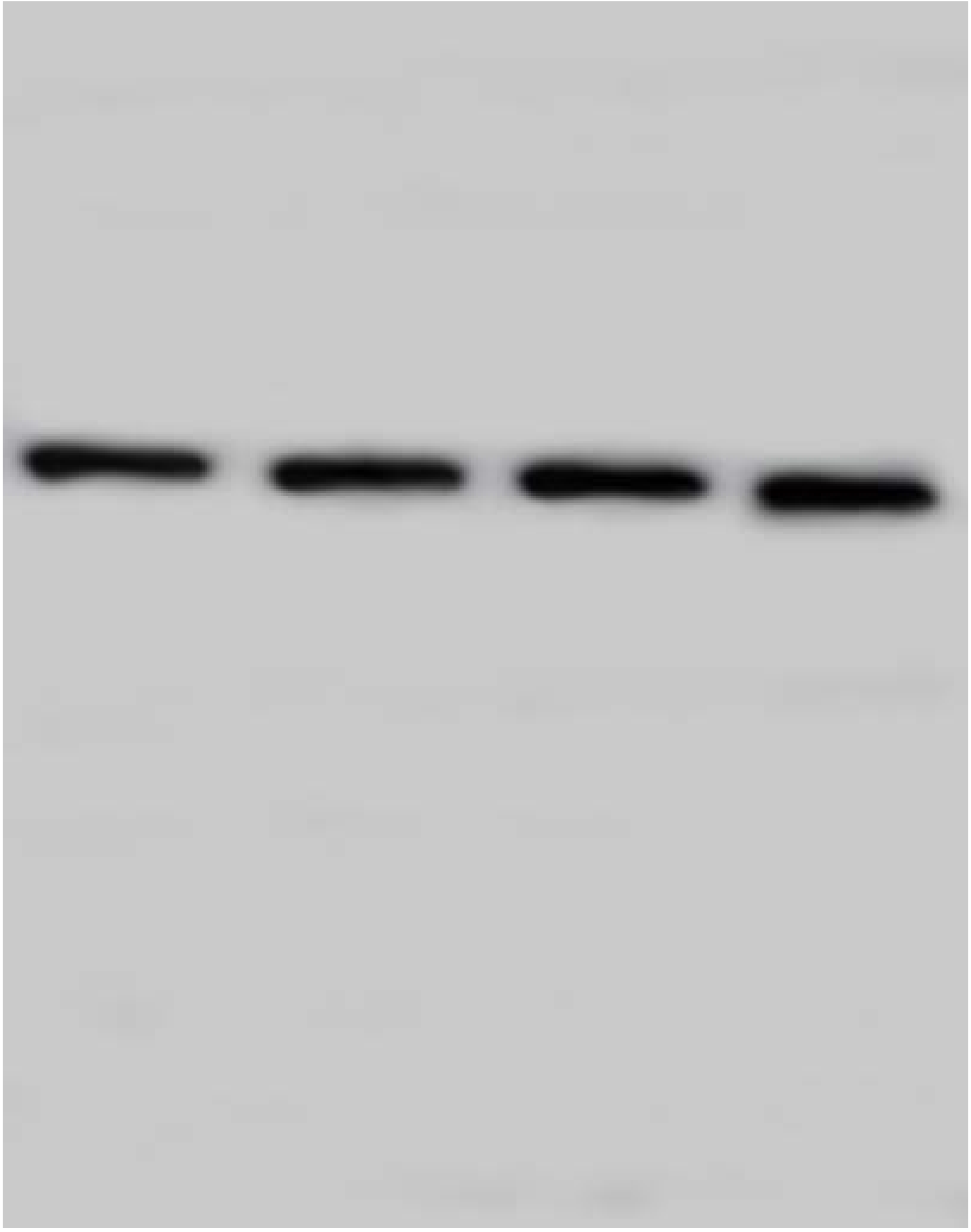

Figure 2G. Beta actin

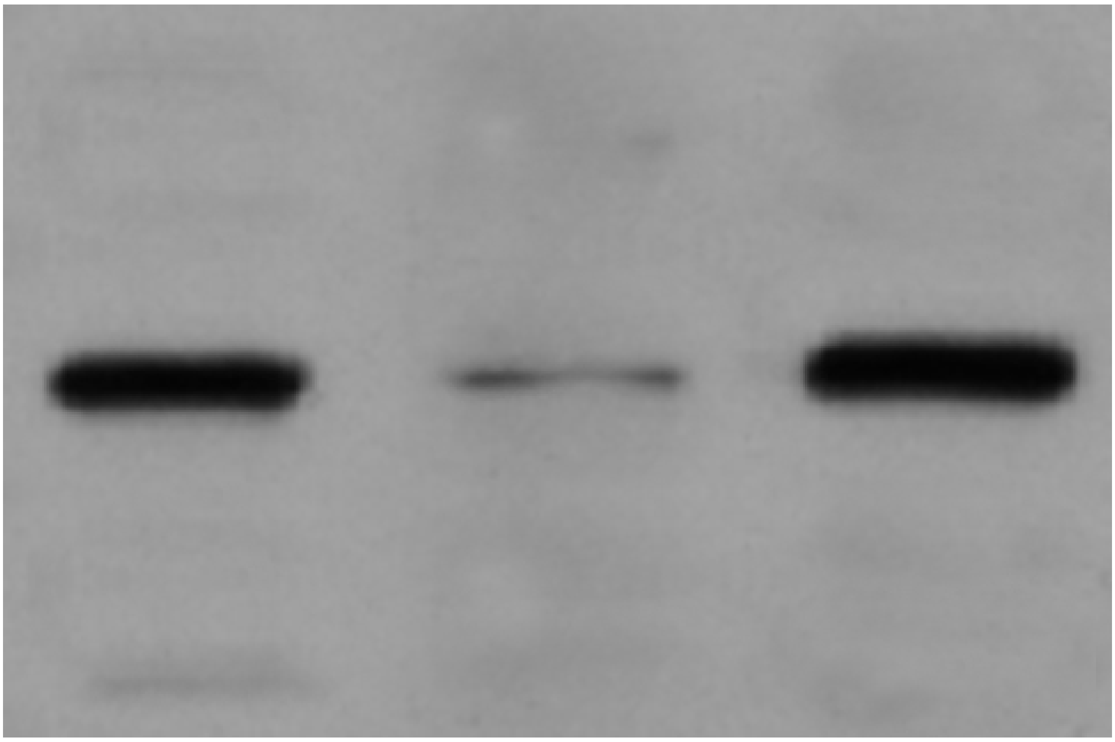

Figure 3L. IL-10.

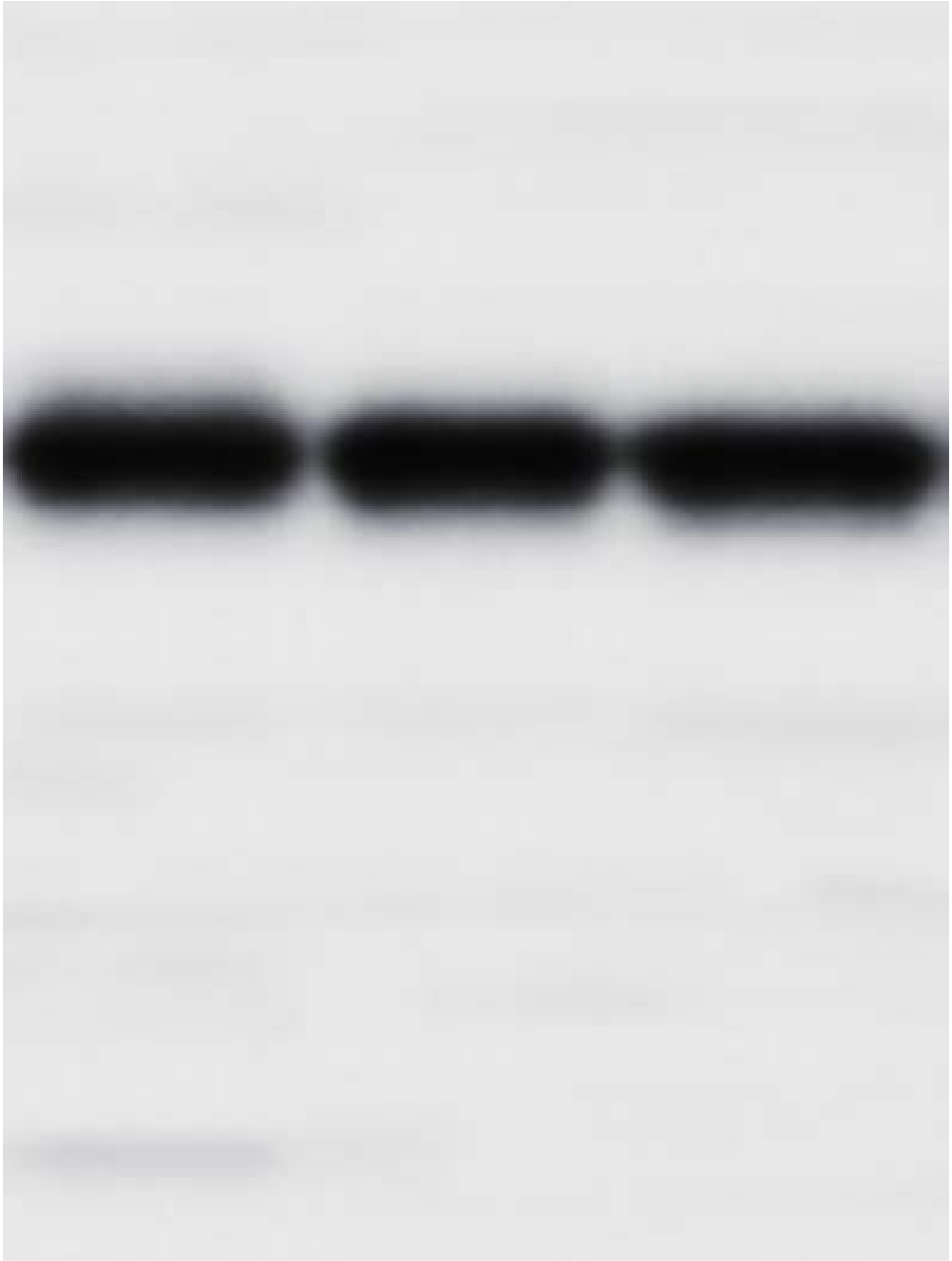

Figure 3L. Beta actin
